# Some Scientific Aspects of Insanity

**Published:** 1891-12-26

**Authors:** 


### SOME SCIENTIFIC ASPECTS OF INSANITY.

### VII.—MELANCHOLIA.

WHEN a patient is suffering from melancholia there is a constant tendency increasing with the malady to disregard external objects, and to become engrossed with the real or imaginary ills under which he is labouring. There is consequently an increasing tendency to disregard the calls of nature, a carelessness amounting in some cases to an antipathy as to food, and a desire varying in intensity at different times of the day and in different patients to be quit of life. Whenever you see a case with undoubted depression of spirits you may assume that there are two factors present, both of them hostile to the patients' interests, namely, a disinclination for food and a disregard for life.

There are other two symptoms which, though of minor consequence compared with the two just mentioned, yet urgently demand attention, they are sleeplessness, and a tendency towards constipation of the bowels.

The lack of tone and muscular energy which is so apparent in the patient is due to a deficiency in the generation and transmission of nerve energy from the gray matter of the central nervous system. Like every other vital function in nature, the production of this nervous energy by the nervous system varies at different times of the day and night. It is low in the early morning. It gradually rises towards noon, when it is at its height. It sinks again in the late afternoon, and rises again from eight to ten in the evening.

During the great function of sleep there is less blood flowing through the brain than at any other time, and as nervous energy is only produced in accordance with the supply of pure blood there is less energy produced during sleep than at any other period of the twenty-four hours. It therefore happens that the period of awakening from sleep in the morning is the time at which melancholics are most depressed, because they have less nervous tone, and it is at this time of the day that most of them commit suicide.

There are many varieties of melancholia just as there are many varieties of the same flower. Yet they all have the one fundamental characteristic—depression of spirits—and they all possess in a more or less prominent degree the four cardinal symptoms, namely, sleeplessness, constipation of the bowels, a disinclination for food, and a disregard for existence.

The varieties of melancholia are named according to the most prominent system of each variety. There is first of all

1. *Simple Melancholia.* It is that form of the disease in which there are fewest bad symptoms or complications. The patient may be very miserable and depressed, he may feel weary of life and unfit to mingle with his friends and acquaintances. He is unable to pursue his ordinary avocations, to carry on a conversation of any length, to read a book, or to read the papers. He imagines that he is unworthy of confidence and respect, or that he is ruined financially or otherwise, or that there is some calamity about to befall him. But he expresses no definite delusions, nor does he threaten to commit suicide or seriously consider the project. He does not refuse to take his food, although he may have little inclination for it. He does not refuse to listen to the arguments or consolations of his friends, although he is not influenced or convinced by them. Such is a case of simple melancholia. The patients usually make a good and speedy recovery under the influence of careful treatment.

2. *Excited Melancholia.* This form of melancholia is often confused with mania because the patient is restless and excited. The great distinction between the two diseases is, of course, that the melancholic is always unhappy or afraid of some impending danger, however great his excitement may be. In the ordinary type of excited melancholia the patient is unable to rest. He cannot sit down, if he does sit he keeps muttering to himself and wringing his hands, most usually he paces up and down the room, with long rapid strides with his head bent down, sometimes wringing his hands, sometimes rubbing the top of his hair with his hands. In this way some of the older melancholics break large patches of the hair on the top of their heads. The excited melancholic has also a tendency to repeat the same story, and in very much the same words to every person he addresses. He often repeats over and over again as he paces up and down his room a phrase such as "Oh dear me, Oh dear me," or "What shall I do?" This form of melancholia requires for its recovery more time, more care, and more vigorous treatment than simple melancholia. The victims have to be most closely watched on account of their great tendency towards self-destruction.

3. *Delusional Melancholia.* Even those labouring under the simpler forms of melancholia are in the strict sense of the term "delusional." But on account of the great prominence of delusions in this variety of the disease it is specially termed delusional. The delusions of melancholic patients are of numerous kinds, but they have all one characteristic in common which is that they are all depreciative of self. It is unnecessary to attempt to enumerate them. Many of them are religious, the patient imagining that his chances of eternal salvation are gone, and that he is doomed to misery both in this life and the next. It is common for them to imagine that they have brought ruin and disaster upon all their friends and acquaintances. A very common delusion which I mention because of its great importance, is one in which the patient expresses his belief that he is being pursued by relentless enemies who are seeking his life, or that he is to be dragged before some judicial tribunal to receive sentence of death. When a patient suffering great terror on account of these beliefs, is being treated by his friends, they commonly ignore the risk of suicide, imagining that a person so much afraid of death would be the last to lay violent hands upon himself. It is, however, the experience of many asylum medical officers that these are just the cases which most frequently commit suicide. There is a milder form of delusional melancholia, but a more chronic form. It is sometimes called hypochondriacal melancholia. In it the patient develops many extraordinary beliefs about the condition of the body. He may imagine that his intestines are blocked, and that he cannot swallow any more food. Such cases are often extremely troublesome, as they persistently refuse to take food, and have to be forcibly fed from year's end to year's end. Others imagine that their bodies are insignificantly small, or on the other hand that they are far too large. The hypochondriacal class of melancholic patients is very frequently afflicted in addition with permanent mental weakness. The presence of delusions in melancholic patients by no means indicates a hopeless termination. The disturbance of intelligence necessarily implied, of course renders them less curable, but the great majority of them under favourable circumstance ultimately recover.

4. *Resistive Melancholia.* Most of the cases of melancholia which we see in asylums, manifest a resistiveness, but there is a class of case which resists everything done for them. They resist their clothes being put on and taken off; they resist going out to walk and returning to the house; they resist the taking of food and interference on the part of the attendants in every matter. Such cases are especially termed resistive. They may be divided into two classes, the recent and younger cases, and the chronic and older cases. The first division only comprises curable cases, but this form of melancholia is on the whole less curable than the others which have been mentioned.

